# Performance evaluation of the high-throughput quantitative Alinity m Epstein-Barr virus assay

**DOI:** 10.1128/spectrum.01507-24

**Published:** 2024-11-15

**Authors:** D. Yitzchak Goldstein, Mark M. Sasaki, Momka Narlieva, Nhi Nhan, Tianxi Liu, April Kegl, Tanjina Akter, Tanisha Dickerson, Eriel Thornton, Patricia Jim, Stephen Young, Danijela Lucic, Julie W. Hirschhorn

**Affiliations:** 1 Department of Pathology, Montefiore Medical Center, Bronx, New York, USA; 2 Molecular Diagnostics of Abbott, Des Plaines, Illinois, USA; 3 Department of Pathology and Laboratory Medicine, Medical University of South Carolina, Charleston, South Carolina, USA; 4 TriCore Reference Laboratories, Albuquerque, New Mexico, USA; Icahn School of Medicine at Mount Sinai, New York, New York, USA

**Keywords:** nucleic acid amplification, DNA, transplant EBV, viral load monitoring, high-throughput diagnostic assay

## Abstract

Molecular testing for Epstein-Barr virus (EBV) infection is a cornerstone of care to prevent adverse outcomes in immunocompromised patients, including transplant recipients. We evaluated the analytical and clinical performance of the quantitative Alinity m EBV assay for plasma sample testing on the fully automated Alinity m platform. Assay lower limit of detection and precision were determined using commercially available panels in plasma. Alinity m EBV detected 100% of panels at 1.3 Log IU/mL, and precision ranged from 1.9% to 5.2% coefficients of variance (SD ≤ 0.14 Log IU/mL). Remnant-de-identified specimens initially tested with the Eurofins Viracor EBV Laboratory Developed Test (LDT) (*n* = 357), ELITech EBV LDT (*n* = 113), University of Washington (UW) LDT (*n* = 151), or Roche cobas EBV assay (*n* = 148) were subsequently tested with the Alinity m EBV assay. Comparison of the Alinity m EBV assay and Eurofins Viracor EBV assay demonstrated a correlation coefficient of 0.762 and mean bias of −0.48 Log IU/mL, comparison of the Alinity m EBV assay with UW LDT demonstrated a correlation coefficient of 0.970 and mean bias of −0.24 Log IU/mL, and comparison of the Alinity m EBV assay with cobas EBV demonstrated a correlation coefficient of 0.964 and mean bias of 0.35 Log IU/mL. The Alinity m EBV assay demonstrated high precision across the analytical measurement range and produced comparable results to the EBV test of record assays. These findings support the utility of the fully automated Alinity m EBV assay in transplant patient management.

**IMPORTANCE:**

Epstein-Barr virus (EBV) infection and reactivation are associated with increased risk for post-transplant lymphoproliferative disorders (PTLD) in transplant recipients with the development of PTLD occurring predominantly within a year of transplant. Quantitative PCR for EBV is used to monitor the viral load of EBV with a negative result as a good negative predictor of PTLD. Nucleic acid amplification tests (NAATs) with high sensitivity and specificity are available on fully automated high-throughput instruments to provide accurate quantitation and improve test result turn-around time. This study evaluates the analytical and clinical performance of one such NAAT, the Alinity m EBV assay.

## INTRODUCTION

Epstein-Barr virus (EBV) is a g-herpesvirus with worldwide prevalence of 90% or greater in the adult population with life-long persistence in B lymphocytes after infection (1, 2). EBV infection is normally asymptomatic but plays a role in the development of a variety of diseases including mononucleosis, multiple sclerosis, and several forms of cancer (3–6). EBV transmission occurs through saliva, blood, sexual contact, or organ transplantation (4, 7–9). Donor-transmitted EBV infection and reactivation of latent EBV in transplant patients are associated with increased risk for post-transplant lymphoproliferative disorders (PTLD) as 13%–33% of solid organ transplant (SOT) recipients and about 3% of allogeneic hematopoietic stem cell transplant (HSCT) recipients develop PTLD (10, 11). In both SOT and HSCT patients, the development of PTLD occurs predominantly within 1 year of transplantation (11, 12). Diagnostic work-up for EBV-associated PTLD includes a physical exam, imaging, endoscopy in case of gastro-intestinal symptoms, histological examination of tissue biopsy, and quantitation of EBV viral load (VL) (11, 13, 14).

Prospective monitoring for EBV in peripheral blood by quantitative PCR is recommended for allogeneic HSCT recipients starting within the first month of allo-HSCT and continued testing once a week for at least 4 months after HSCT (11). A negative EBV result is a good negative predictor of PTLD early after transplant in seronegative children at high risk (15). As such, accurate VL quantitation is important for treatment planning and evaluation of treatment response. EBV nucleic acid amplification tests (NAATs) have high sensitivity and specificity and provide accurate quantitation of VL. Here, we evaluate the analytical and clinical performance of the Alinity m EBV (Des Plaines, IL, USA) assay in the reference and clinical lab of a large tertiary care hospital.

## MATERIALS AND METHODS

### Study design and specimens

Performance characteristics of the Alinity m EBV assay were assessed in comparison to the following clinical tests of record: ELITech EBV Laboratory Developed Test (LDT) (ELITech Group, Logan, UT, USA) run on the Abbott *m*2000*sp/rt* (Molecular Diagnostics of Abbott, Des Plaines, IL, USA) and performed at the Medical University of South Carolina (MUSC) Department of Pathology and Laboratory Medicine (Charleston, SC, USA), EBV LDT performed at the University of Washington Medicine Clinical Virology Laboratory (Seattle, WA, USA), and Roche cobas EBV run on the cobas 6800 system performed at TriCore Reference Laboratories (Albuquerque, NM, USA), as well samples sent for comparison to a large national reference laboratory utilizing an EBV LDT at Eurofins Viracor (Lexana, KS, USA).

Sample testing with the Alinity m EBV assay was performed at Montefiore Medical Center, MUSC, and Molecular Diagnostics of Abbott (Des Plaines, IL, USA). At MUSC, the study protocol was considered a quality improvement project and was not subject to Institutional Review Board (IRB) review per MUSC operating procedure.

A total of 357 residual de-identified plasma samples were initially tested with the Eurofins Viracor EBV assay and subsequently tested fresh or after storage at −70°C with the Alinity m EBV assay. All specimens were anonymized before study initiation, and a coded identification number containing no patient identifier was assigned to each remnant specimen. A total of 120 surplus remnant de-identified plasma specimens that were initially tested with the ELITech EBV LDT run on *m*2000*sp/rt* were tested after storage at −70°C with the Alinity m EBV assay. A total of 170 surplus remnant plasma specimens tested with EBV LDT were procured from the University of Washington Medicine Clinical Virology Laboratory and tested after storage at −70°C with the Alinity m EBV and cobas EBV assays.

### Molecular assays

The Alinity m EBV assay (Abbott Molecular Inc., Des Plaines, IL, USA) is a real-time dual-target (GP350 and EBNA1) PCR assay that is run on the Alinity m platform with a quantitative range from 1.70 to 8.30 Log IU/mL.

The EBV quantitative assay from Eurofins Viracor EBV LDT (test 4500, Eurofins Viracor, Lenexa, KS, USA) has a quantitative range from 1.69 to 8.23 Log IU/mL.

The ELITech EBV LDT assay (ELITechGroup, Puteaux, France) is a real-time PCR test run on the *m*2000 platform with a quantitative range from 2.70 to 6.70 Log IU/mL.

The cobas EBV assay (Roche, Indianapolis, IN, USA) is a real-time dual-target (EBNA1 and BMRF) PCR assay run on the cobas 6800 platform with a quantitative range from 1.54 to 8.00 Log IU/mL.

### Analytical performance assessment

Alinity m EBV assay sensitivity was assessed by testing 20 replicates prepared at 1.30 Log IU/mL by diluting a commercially available EBV verification panel (Exact Diagnostics, Fort Worth, TX, USA) with pooled and confirmed negative EBV plasma. Linearity was established across the range of EBV concentrations from 2.00 Log IU/mL to 7.70 Log IU/mL using a commercially available EBV panel in plasma (Exact Diagnostics, Fort Worth, TX, USA) and the 1.48 Log IU/mL panel prepared by diluting one of the commercially available EBV panel in negative plasma. Alinity m EBV assay precision was evaluated by testing panels ranging from 2.70 to 6.70 Log IU/mL across two sites.

Alinity m EBV reproducibility was assessed across two sites evaluating the performance of the assay quality controls (QC) [high positive control (HPC) ranging from 4.97 to 5.11 Log IU/mL and low positive control (LPC) ranging from 2.91 to 3.03 Log IU/mL]. A total of 41 replicates were tested each for HPC and LPC.

### Instrument turn-around times

Onboard and processing turn-around times (TATs) of the Alinity m system were evaluated based on the automatic documentation by Alinity m of timepoints for sample loading, sample aspiration and result reporting.

### Statistical analysis

All analyses were performed using PC SAS (version 9.4) software (SAS, Cary, NC, USA). Relationships between quantitative values were studied by means of Deming regression. Bland-Altman analysis was performed to evaluate the differences in quantification between the assays. The following precision (variance component) analysis was performed for Alinity m EBV precision data.

The following analysis was performed for each instrument and each panel member: the PROC MIXED procedure with the MIVQUE0 option in SAS was used to produce variance components for the model used in the analysis. The point estimates of the means, SD, and % coefficients of variance (CV) were reported. The SD and %CV were estimated for the within-day component, the between-day component, and the between-site component for each instrument and each panel member. All the effects were considered as random for the analyses. Any negative variance components were set to zero for these calculations. Variance components were estimated based on the random effects ANOVA model: *Y* = mean + site + day + error. The total assay variability was defined as the sum of the within-day (residual error) component, the between-day component, and the between-site component estimates of variability. The following statistics were reported: *N*, mean, within-day SD and %CV, between-day SD and %CV, between-site SD and %CV, and total SD and %CV.

## RESULTS

### Analytical performance

**Fig 1:**
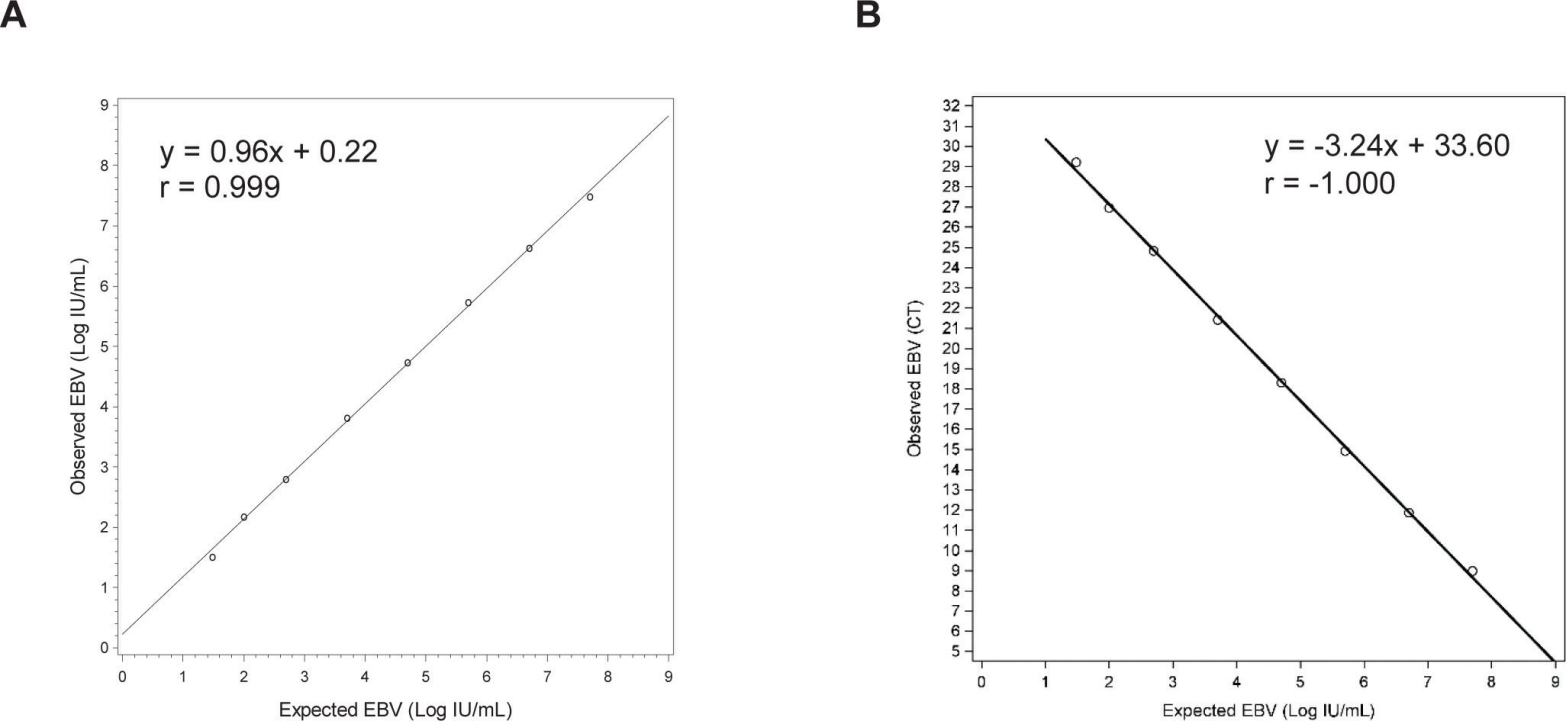
Analytical performance of the Alinity m EBV assay. Linearity was established across the range of EBV concentrations from 1.48 Log IU/mL to 7.7 Log IU/mL using commercially available dilution panels in plasma. (**A**) Target concentration versus observed concentration. (**B**) Target concentration versus observed Ct.

Alinity m EBV assay detected 100% (20/20) of replicates at 1.30 Log IU/mL, the claimed limit of detection of the assay. Linearity of Alinity m EBV using a commercially available verification panel had a correlation coefficient (*r*) of 0.999 (Deming regression equation, *y* = 0.96*x* + 0.22) with inverse correlation with Ct (Fig. 1B, Deming regression equation, *y* = −3.24 + 33.60, *r* = −1.000). In the precision analysis, the total CV ranged from 1.9% to 5.2% with total SD ≤ 0.14 Log IU/mL (Table 1).

**TABLE 1:** Precision and clinical reproducibility of the Alinity m EBV assay

Expected concentration (Log IU/mL)	Mean observed concentration (Log IU/mL)	Δ (Log IU/mL)	Within day		Between day		Between sites		Total^*a*^	
			SD	%CV	SD	%CV	SD	%CV	SD	%CV
2.70	2.39	−0.31	0.11	4.7	0.05	2.1	0.00	0.0	0.12	5.2
3.70	3.48	−0.22	0.08	2.3	0.07	2.1	0.00	0.0	0.11	3.1
4.70	4.47	−0.23	0.08	1.7	0.11	2.5	0.00	0.0	0.14	3.1
5.70	5.53	−0.17	0.07	1.3	0.09	1.7	0.00	0.0	0.12	2.2
6.70	6.54	−0.16	0.08	1.2	0.08	1.3	0.05	0.7	0.12	1.9

^a^ Total includes within-day, between-day, and between-site components.

Alinity m EBV reproducibility was assessed testing 41 replicates of HPC and LPC across two sites. The total %CV for HPC and LPC were 3.7% and 6.6%, respectively, and the total SD were 0.19 Log IU/mL and 0.20 Log IU/mL, respectively (Table 2).

**TABLE 2:** Reproducibility testing of Alinity m CMV quality controls across two laboratories

Panel member^*a*^	*n*	Target conc. (Log IU/mL)	Mean conc. (Log IU/mL)	Within-day component		Between-day component		Between-site component		Total	
				SD	%CV	SD	%CV	SD	%CV	SD	%CV
HPC	41	4.97–5.11	5.04	0.09	1.8	0.00	0.0	0.16	3.3	0.19	3.7
LPC	41	2.91–3.03	3.07	0.11	3.7	0.00	0.0	0.17	5.5	0.2	6.6

^*a*^ HPC, high positive control; LPC, low positive control.

### Clinical performance

**Fig 2:**
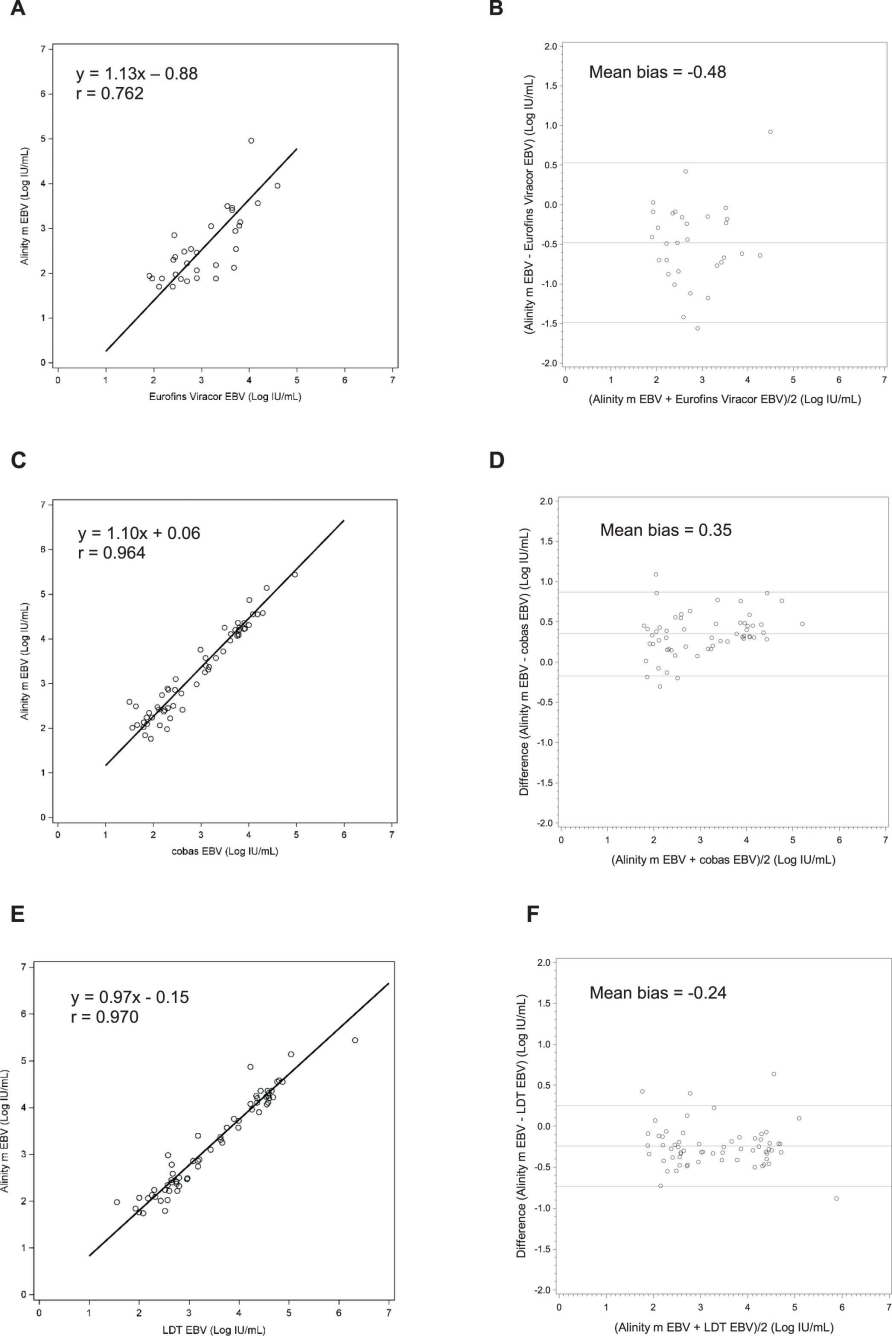
Clinical performance of the Alinity m EBV assay compared with Eurofins Viracor EBV, cobas EBV, and LDT EBV assays with plasma samples. Deming regression of (**A, C, and E**) EBV levels showing correlation between the Alinity m EBV and the Eurofins Viracor EBV assays. (**B, D, and F**) Bland-Altman analysis showing mean bias between the Alinity m EBV and the Eurofins Viracor EBV assays. Solid line indicates mean bias, and dotted lines indicate ±1.96*x* SD.

Three-hundred fifty-seven plasma (*n* = 357) specimens were tested with the Alinity m EBV assay and the Eurofins Viracor EBV PCR LDT (Table 3). The overall observed agreement between the two assays was 88.0% (315/357). The overall sensitivity for the Abbott Alinity m EBV assay was found to be 97.1% (68/70). Positive percent agreement (PPA) including all samples, which were identified as below lower limit of quantitation (LLOQ), was 62.4% with Alinity EBV identifying 36 samples as being <LLOQ, which were previously identified as negative by the Viracor assay. Negative percent agreement (NPA) was 99.2% (246/248). Of the 357 specimens tested, 31 were within the quantifiable range of both assays. The correlation coefficient was 0.762 (Deming regression equation, *y* = 1.13*x* − 0.88), and mean bias was −0.48 Log IU/mL (Bland-Altman analysis, Alinity m EBV – Eurofins Viracor EBV; Fig. 2A and B).

**TABLE 3:** Agreement between the Alinity m EBV assay and the Eurofins Viracor EBV PCR assay (*n* = 357 plasma samples)

		Eurofins Viracor EBV			
		Not detected	<LLOQ^*a*^	Quantitated	Total
Alinity m EBV	Not detected	246	2	0	248
	<LLOQ^*a*^	36	10	27^*c*^	73
	Quantitated	5^*b*^	0	31	36
	Total	287	12	58	357

^*a*^ LLOQ, lower limit of quantitation. LLOQ used here is the higher LLOQ between Alinity m EBV and Eurofins Viracor EBV.

^*b*^ Five specimens not detected by Viracor EBV had a range of 1.89 to 2.58 Log IU/mL on Alinity m EBV.

^*c*^ Twenty-seven specimens < LLOQ by Alinity m EBV had a range of 1.72 to 3.15 Log IU/mL.

A total of 148 EBV-positive specimens were tested with the Alinity m EBV and cobas EBV assays (Table 4). The overall observed agreement between the two assays was 83.1% (123/148). The overall sensitivity was found to be 98.0% (96/98). PPA including all samples, which were identified as LLOQ, was 80.7%, and NPA was 93.1%. Of the 148 specimens tested, 57 were within the quantitative range for both assays. The correlation coefficient was 0.964 (Deming regression equation, *y* = 1.10*x* + 0.06), and mean bias was 0.35 Log IU/mL (Bland-Altman analysis, Alinity m EBV – cobas EBV; Fig. 2C and D).

**TABLE 4:** Agreement between the Alinity m EBV assay and the Roche cobas EBV assay (*n* = 148 plasma samples)

		Roche cobas EBV			
		Not detected	<LLOQ^*a*^	Quantitated	Total
Alinity m EBV	Not detected	27	2	0	29
	<LLOQ^*a*^	23	32	2^*c*^	57
	Quantitated	0	8^*b*^	54	62
	Total	50	42	56	148

^*a*^ LLOQ used here is the higher LLOQ between Alinity m EBV and Roche cobas EBV.

^*b*^ Eight specimens < LLOQ by cobas EBV had a range of 1.74 to 2.59 Log IU/mL on Alinity m EBV.

^*c*^ Two specimens < LLOQ by Alinity m EBV were quantitated at 1.83 and 2.51 Log IU/mL by cobas EBV.

One-hundred fifty-one (*n* = 151) EBV-positive specimens were tested with Alinity m EBV and LDT EBV assays (Table 5). The overall observed agreement between the two assays was 80.8% (122/151). Of the 151 specimens tested, 64 were within the quantitative range for both assays. The correlation coefficient was 0.970 (Deming regression equation, *y* = 0.97*x* − 0.15), and the mean bias was −0.24 Log IU/mL (Bland-Altman analysis, Alinity m EBV – LDT EBV; Fig. 2E and F).

**TABLE 5:** Agreement between the Alinity m EBV assay and the LDT EBV assay (*n* = 151 plasma samples)

		LDT EBV			
		Not detected	<LLOQ^*a*^	Quantitated	Total
Alinity m EBV	Not detected	0	28	1^*c*^	29
	<LLOQ^*a*^	0	35	23	58
	Quantitated	0	1^*b*^	63	64
	Total	0	64	87	151

^*a*^ LLOQ used here is the higher LLOQ between Alinity m EBV and LDT EBV.

^*b*^ One specimen <LLOQ by LDT EBV was quantitated at 1.99 Log IU/mL on Alinity m EBV.

^*c*^ One specimen not detected by Alinity m EBV was quantitated at 1.70 Log IU/mL by LDT EBV.

A total of 113 plasma specimens were tested with the ELITech EBV assay run on *m*2000*sp/rt* and Alinity m EBV assay (Table 6). The overall observed agreement between the two assays was 82.3% (93/113), PPA was 72.5% (37/51), and NPA was 90.3% (56/62). Alinity m EBV identified 14 samples as being <LLOQ, which were previously identified as negative by the ELITech EBV assay. Of the 113 specimens tested, only one was within the quantitative range for both assays.

**TABLE 6:** Agreement between the Alinity m EBV assay and the ELITech EBV assay (*n* = 113 plasma samples)

		ELITech EBV			
		Not detected	<LLOQ^*a*^	Quantitated	Total
Alinity m EBV	Not detected	56	6	0	62
	<LLOQ^*a*^	14	26	1^*c*^	47
	Quantitated	0	3^*b*^	1	4
	Total	70	35	8	113

^*a*^ LLOQ used here is the higher LLOQ between Alinity m EBV and ELITech EBV.

^*b*^ Three specimens < LLOQ ELITech EBV had a range of 2.87 to 3.01 Log IU/mL on Alinity m EBV.

^*c*^ One specimen < LLOQ by Alinity m EBV was quantitated at 3.00 Log IU/mL by ELITech EBV.

### Instrument TAT

The Alinity m system allows for random and continuous processing of samples tested with different assays side by side. Observed median onboard TAT from placement of specimen on the Alinity m to result reporting for Alinity m EBV was 2 hours and 35 minutes (ranging from 2 hours 6 minutes to 4 hours 54 minutes).

## DISCUSSION

This multi-site study demonstrated that the Alinity m EBV assay performed with 100% sensitivity at 1.30 Log IU/mL, the claimed limit of detection and high precision across the dynamic range. Total %CV was ≤5.2%, and total SD was ≤0.14 Log IU/mL for all panel members tested for precision. On average, the quantitation was 0.22 Log IU/mL lower than the manufacturer-assigned target values of the verification panel. This difference in quantitation was observed at multiple sites and instruments. A different verification panel from the same manufacturer was used for the linearity study that included panel members with the same value assignment as those used in the precision study. These samples quantitated 0.03 Log IU/mL higher than the manufacturer-assigned target values, suggesting that the difference in quantitation observed in the precision study may be due to the manufacturer’s value assignment acceptance criteria for the panel members. Alinity m EBV also demonstrated excellent reproducibility for LPC and HPC with a total %CV of 6.6% and 3.7% and total SD of 0.20 Log IU/mL and 0.19 Log IU/mL, respectively.

Clinical performance of the Alinity m EBV assay was compared with two commercially available assays and two laboratory-developed tests (Table 7). Specimens tested with the Eurofins Viracor EBV assay were shipped to a reference site for testing. OPA was 88.0%, PPA was 62.4%, and NPA was 99.2%. Forty-one (*n* = 41) specimens that were not detected by the Viracor EBV assay were detected by the Alinity m EBV assay; of these, 36 specimens were detected <LLOQ and 5 specimens were quantitated between 1.72 and 3.15 Log IU/mL. Mean bias observed between the Alinity m EBV and Viracor EBV assays was −0.48 Log IU/mL.

**TABLE 7:** Summary of clinical performance of the Alinity m EBV with comparator assays

	Viracor EBV	EBV LDT	Cobas EBV	ELITech EBV
Correlation coefficient (*r*)	0.762	0.970	0.964	N/A^*a*^
Mean bias (Log IU/mL)	−0.48	−0.24	0.35	N/A
Overall agreement (%, *n*/*N*)	88.0% (315/357)	80.8% (122/151)	83.1% (123/148)	82.3% (93/113)

^*a*^ N/A, not available.

The overall percent agreement between the Alinity m EBV and ELITech EBV assays was 82.3%, and PPA was 72.5%, and NPA was 90.3%. Discordant results were unable to be resolved due to insufficient volume. Deming regression and Bland-Altman analyses were not possible as only 1 of 113 samples were within the quantifiable range of both assays.

Comparable differences in quantitation were also observed between the Alinity m EBV and LDT EBV assays with a mean bias of −0.24 Log IU/mL. Strong correlation was observed between the two assays (*r* = 0.970). Of the 151 total EBV-positive specimens by LDT EBV, 29 were not detected by Alinity m EBV. Of the 29 specimens, 28 were quantitated <LLOQ with LDT EBV and 1 was quantitated at 1.70 Log IU/mL. Twenty-seven (*n* = 27) of the 29 were not detected on a third comparator assay and 2 were detected <LLOQ.

Comparison between the Alinity m EBV and cobas EBV assays demonstrated strong correlation (*r* = 0.964) with Alinity m EBV quantitating 0.35 Log IU/mL higher than cobas EBV. Of the 50 samples not detected by cobas EBV, 23 were detected <LLOQ by Alinity m EBV, which were also detected on a third comparator assay.

Sample storage conditions prior to testing may have contributed to difference in quantitation of specimens tested on Alinity m EBV and the comparator assays. Fresh and frozen specimens were tested on Viracor EBV but were stored at −70°C prior to testing with Alinity m EBV. Fresh specimens were tested on the LDT EBV assay but were stored at −70°C prior to testing with Alinity m EBV. Differences in detection and quantitation between Alinity m EBV and cobas EBV may be attributed to assay design and specimen storage condition as specimens were stored at −70°C prior to testing for both assays but those for cobas EBV testing underwent an additional freeze/thaw cycle. Insufficient volume prevented testing samples on the primary platform following storage to determine if this was the case. Calibration strategy may contribute to the differences in quantitation between Alinity m EBV that uses calibrators standardized to the World Health Organization (WHO) international standard and assays that establish calibration curves using commercially available panels, which have a larger range of quantitation for acceptance. Interlaboratory differences have been observed when testing WHO EBV international standard and prepared reference standards performing EBV testing using commercially available reagents; as such, it is recommended that patients should be monitored using the same sample type and the same assay in a single laboratory (15–17).

Current procedures for testing on *m*2000*sp/rt* lead to an average TAT of over 5 hours from sample loading to results reporting. Eurofins Viracor EBV testing is performed as a send out to an external testing laboratory with an average total TAT of 40 hours from the sample received to results reported. The Alinity m system with random and continuous access capabilities resulted in a median TAT of less than 3 hours across the two laboratories, which allows for same day reporting of results. Our findings support the utility of the Alinity m EBV assay in transplant patient management. Clinical decision making relies on precise quantitation of EBV, and faster reporting of test results with fully automated systems such as Alinity m platform is expected to have a significant impact on transplant patient care.
